# Gut Microbiota Modulation on Intestinal Mucosal Adaptive Immunity

**DOI:** 10.1155/2019/4735040

**Published:** 2019-10-03

**Authors:** Li Wang, Limeng Zhu, Song Qin

**Affiliations:** ^1^Hepatology Department, Infectious Disease Hospital of Yantai, 62 Huanshan Road, Zhifu District, Yantai 264001, China; ^2^Institute of Processing Engineering, University of Chinese Academy of Sciences, 1 North 2nd Street, Zhongguancun, Haidian District, Beijing 100049, China; ^3^Yantai Institute of Costal Zone Research, Chinese Academy of Sciences, 17 Chunhui Road, Laishan District, Yantai 264003, China

## Abstract

The mammalian intestine harbors a remarkable number of microbes and their components and metabolites, which are fundamental for the instigation and development of the host immune system. The intestinal innate and adaptive immunity coordinate and interact with the symbionts contributing to the intestinal homeostasis through establishment of a mutually beneficial relationship by tolerating to symbiotic microbiota and retaining the ability to exert proinflammatory response towards invasive pathogens. Imbalance between the intestinal immune system and commensal organisms disrupts the intestinal microbiological homeostasis, leading to microbiota dysbiosis, compromised integrity of the intestinal barrier, and proinflammatory immune responses towards symbionts. This, in turn, exacerbates the degree of the imbalance. Intestinal adaptive immunity plays a critical role in maintaining immune tolerance towards symbionts and the integrity of intestinal barrier, while the innate immune system regulates the adaptive immune responses to intestinal commensal bacteria. In this review, we will summarize recent findings on the effects and mechanisms of gut microbiota on intestinal adaptive immunity and the plasticity of several immune cells under diverse microenvironmental settings.

## 1. Introduction

The mammalian intestine harbors a vast microbiota, fundamental for the development and maintenance of the host immune system serving as an important epigenetic system [1]. A range of microbiota derivatives and metabolites can modulate host intestinal immune functions by influencing various cell types, including intestinal epithelial cells (IECs), mononuclear phagocytes, innate lymphoid cells (ILCs), and B and T lymphocytes [2]. The intestinal homeostasis requires a dexterously regulated network of the immune cells and their interplay with symbionts. The intestinal innate and adaptive immunities initiated by commensal microbiota coordinate protection of the host from invasion by foreign pathogens and intestine homeostasis [3, 4]. The intestinal adaptive immunity induced by intestinal resident microbiota, associated with differentiation of CD4^+^ T cells and IgA-producing B cells in Peyer's patches (PPs) and lamina propria (LP), and intestinal epithelial lymphocytes (IELs) play a critical role in maintaining immune tolerance towards symbiotic bacteria, integrity of intestine barrier, and gut homeostasis [5]. A critical time window shortly after birth is regarded as vital for the development of gut-associated lymphoid tissue (GALT) in addition to differentiation and maturation of T cells and B cells. This phenomenon is of high consequence as the sequence and composition of colonized intestinal microbiota in infants influence the vaccine efficacy [6].

Microbiota dysbiosis increases host susceptibility to various immune, inflammatory, and allergy disorders of intestine and remote organs [7, 8]. It is shown that restoration of microbiota dysbiosis induced by antibiotics or other factors through administration of probiotics or fecal microbiota transplantation (FMT) of intestinal commensal bacterial species, such as *Escherichia coli* and *Lactobacillus johnsonii*, restores the suppressed adaptive immune functions in human and mice [9–11]. The microbiota and their derived small-molecule metabolites regulate immune cells through direct and indirect effects at the cellular and molecular level. For example, short-chain fatty acids (SCFAs)—a gut microbiota metabolite—influence the fate of immune cells through direct epigenetic modification-induced alteration of metabolism via inhibition of histone deacetylase (HDAC) [12–14].

The fact that intestinal microbiota is a key orchestrator in cancer immunotherapy has recently gained acceptance [15, 16]. Intestinal dysbiosis contributes to primary resistance towards immune checkpoint inhibitors (ICIs) [17]. Studies have revealed that certain species exhibit evident effects on antitumor efficacy of ICIs via promoting dendritic cell (DC) and CD8^+^ T cell functions and inhibiting regulatory T cells (Tregs) [18, 19]. For instance, melanoma does not respond to cytotoxic T lymphocyte antigen 4 (CTLA-4) blockade in antibiotic-treated or germ-free mice (GF), and this defect is rescued by administration of *Bacteroides fragilis*, *B. fragilis* polysaccharides, or *B. fragilis*-specific T cells [20].

The precise effect mechanisms underlying the effect of defined intestinal microbiota and metabolites on specific immune cell function still need to be elucidated. This review is aimed at highlighting the results of recent studies on the effects and mechanisms of intestinal microbiota and derived metabolites and signals on the development as well as differentiation of intestinal adaptive immune cells. This review also pays special emphasis on the induction of intestinal immune tolerance that is also associated with the intestinal innate immunity.

## 2. Composition of the Intestinal Adaptive Immunity

The intestinal mucosal immune system involves GALT that mainly includes aggregated lymphoid follicles, such as the PP, effector lymphocytes in LP, intraepithelial lymphocytes (IELs), and a series of molecules. The intestinal homeostasis requires a delicate balance between the effector T cells and the regulatory T cells, which mediate immune response to pathogens and confine excessive immune activation. Secretory immunoglobulin A (sIgA) that binds to commensal and invading microbes contributes to the homeostasis of intestinal mucosal immunity and barrier function [21]. In addition, intestinal innate immunity plays an important role in contributing immune tolerant features of adaptive immunity. Recent research has demonstrated that group 3 innate lymphoid cells (ILC3s) regulate interactions between T follicular helper (Tfh) cells and B cells, in a steady state, to limit mucosal IgA responses [22, 23]. Furthermore, and under physiological conditions, the IECs promote generation of T cell responses against the resident microbiota through endocytosis of antigens from commensal bacteria, such as proteins from segmented filamentous bacteria (SFB) [23].

## 3. Gut Microbiota and CD4^+^ T Cell Differentiation

CD4^+^ T cells are mainly located in the intestinal LP, most of which are effector or memory T cells. CD4^+^ T cell responses vary greatly depending on the niche of colonization, antigen type, and metabolic property of gut microbiota, which results in the generation of distinct T cell subsets and the functional plasticity of certain T cell subsets [24]. Upon activation by microbiota, antigens are presented by antigen-presenting cells (APCs) such as dendritic cells (DCs), and CD4^+^ T cells differentiate into Tregs and various T helper (Th1) cells such as IFN-*γ*, IL-4, B cell regulating, and IL-17 producing Th1, Th2, Tfh, and Th17 cells, respectively [25, 26]. Th17 cells and Treg cells are the extensively studied subsets of T cells constituting a large proportion of the effector cells. The imbalance between the cells of these two subsets results in various disorders ranging from inflammatory and autoimmune diseases to infection and cancer [27]. A recent study indicates that the transfer of inflammatory bowel disease (IBD) microbiota into GF mice increases the number of intestinal Th17 cells and Th2 cells and decreases retinoic acid-related orphan receptor *γ*t (ROR*γ*t)^+^ Treg cells as compared with transferred microbiota from healthy donors. Gut microbiota-derived SCFAs have been demonstrated to regulate T cell differentiation in a concentration and immunological milieu-dependent manner. For instance, Kespohl et al. have reported that although a lower butyrate concentration can facilitate Treg differentiation under steady-state conditions *in vitro* and *in vivo*, higher concentrations of butyrate induce the expression of the transcription factor T-bet in all the investigated T cell subsets resulting in the differentiation of IFN-*γ*-producing Tregs or conventional T cells [28]. In contrast, Chen et al. have shown that butyrate controls the T cell capacity in the induction of colitis through differential regulation of Th1 and Th17 cell differentiation and promotion of IL-10 production [14].

Proportions of Th17 and ROR*γ*t^+^ Treg cells induced by different microbiota are predictive of human disease status and contribute to disease severity in the Rag1-/- colitis model suggesting a general mechanism that enlightens the contributions of microbiota in IBD pathogenesis [29]. Han et al. have demonstrated that in patients of acute graft-versus-host disease (aGVHD), the microbiota was depleted of Clostridia (e.g., the Lachnospiraceae and Ruminococcaceae families) and enriched for Gammaproteobacteria (e.g., the Enterobacteriaceae family) as compared with the non-aGVHD group. Moreover, the Treg/Th17 ratio positively correlates with the relative abundance of intestinal Lachnospiraceae and Ruminococcaceae. Furthermore, the Treg/Th17 ratio and Lachnospiraceae/Ruminococcaceae ratio are correlated with the level of acetylated H3 in CD4^+^ T cells [30]. Though functions of Treg and Th17 are prominently diverse, they share several important features during differentiation and function [31, 32].

### 3.1. Effects of Gut Microbiota and Metabolites on Th17 Cell Plasticity

Th17 cells are critical in the defense against pathogens, especially in the case of extracellular bacterial and fungal infections. In response to SFB colonization, naïve CD4^+^ T cells can migrate to LP of the small intestinal and differentiate into IL-17A producing Th17 cells. IL-17A, IL-17F, and IL-22 produced by Th17, in turn, stimulate IECs to produce antimicrobial peptides (AMPs) [33] and maintain integrity of the intestinal barrier in a noninflammatory manner [34]. In addition, Th17 cells are crucial for the production of T cell-dependent (TD) high-affinity bacteria-specific IgA [35]. Excessive activation of Th17 cells can result in autoimmune diseases [36]. Microbiota, especially SFB, influences the differentiation of Th17 cells [37]. Transcription factor ROR*γ*t is essential in the differentiation of IL-17 producing CD4^+^ Th17 and Th1/17 cells that coproduce IL-17 and IFN-*γ*. It has been demonstrated that T cell antigen receptors (TCR) specific for SFB-encoded peptides promote CD4^+^ T cell differentiation into ROR*γ*t-expressing Th17 cells, even if the SFB-colonized mice also harbors in their intestine Listeria monocytogenes—a strong Th1 cell inducer [38]. Nevertheless, a recently published data shows that the SFB responding T cells comprise of a heterogeneous population including Th1, Th17, and Tfh cells proven by single cell RT-PCR analysis [39].

The dichotomous nature of Th17 cells also enables them to be pathogenic drivers, especially in IBD [40]. Intestinal Th1 and Th17 cells also play an indispensable role in microbiota-promoted colorectal cancer development. A study has revealed that proportions of Th1 and Th17 cells and cytokines, IL-17A, IL-22, and IL-23A, produced by Th17 cells are higher in conventional mice than in GF mice when fed with stool from patients with colorectal cancer [41]. Furthermore, the Th1/Th17 balance has turned out to be associated with the prognosis of patients with colorectal tumors. Th1 cell-mediated anticancer responses are associated with better outcomes, whereas Th17 cell-mediated responses are associated with worse outcomes implicating that specific members of the gut microbiota promote IL-17A production, thereby contributing to carcinogenesis [42].

The role of intestinal microbiota in modulating response efficacy to ICI therapy has drawn considerable attention in recent years. In patients with melanoma, relative abundance of the Ruminococcaceae family is increased in responders who receive anti-PD1 therapy, while the poor responders exhibit increased counts of ROR*γ*t^+^ Th17 cells in tumors. FMT in GF mice has demonstrated that activation of Th17 cells and the polarization of IL-17A production in the tumor microenvironment are essentially mediated by components of the microbiota [43]. A study has shown that the number of pathogenic IL-17A^+^ IFN-*γ*^+^ and IL-22^+^ IFN-*γ*^+^ Th17 cell subsets is negatively regulated by intestinal HLA-DR-expressing-NKp44^+^-ILC3s in IBD patients [44]. IL-6 has been shown to trigger Th17 cell differentiation via STAT3 activation, thus promoting inflammation in IBD patients, whereas leukemia inhibitory factor (LIF)—a cytokine of the IL-6 family—effectively inhibits Th17 accumulation and promotes damaged intestinal epithelium repair [45]. In addition, the role of IL-6 in Th17 lineage priming and differentiation is tissue specific [46] and involves reversible induction of Th1-to-Th17 cell transdifferentiation in the intestine [47]. Smad7 is an intracellular inhibitor of the transforming growth factor-beta (TGF-*β*) signaling, whose expression in colorectal cancer-infiltrating Th17 cells increases tumor necrosis factor-*α* (TNF-*α*) and interferon-*γ* (INF-*γ*) expression and decreases IL-17A expression that is responsible for effective killing of cancer cells [48]. Therefore, the plasticity of Th17 cells is dependent on the physiological or pathogenic cytokine milieu *in vivo* and interactions between different immune cells. However, precise driving factors for this shift remain less understood. Therefore, the development of therapeutic strategies targeting the IL-17 pathway needs to be carefully evaluated lest the potential side effects may hijack the beneficial functions of Th17 cells. The effects of SFB on Th17 cells in humans need further investigation, as it is not well known in human intestine.

### 3.2. Treg Cell Induction by Microbiota and Interactions with Other Immune Cells

Within the intestine, forkhead box P3 transcription factor- (Foxp3-) expressing Treg cells are primarily located in the LP [49] and have a fundamental role in immunological tolerance towards commensal microbiota. The Foxp3+ Treg repertoire is heavily influenced by the microbiota composition [50]. Upon migration to the epithelium, Tregs lose their Foxp3 expression and convert to effective CD4^+^ T cell in a microbiota-dependent manner [49].

Gut-derived Foxp3^+^ Treg cells are distinct from those in other organs and have gut-specific phenotypes and functions. Symbiotic bacteria, for instance, *Clostridium* species clusters [51, 52] and *Bacteroides fragilis* and its polysaccharide A [53], can facilitate the expansion and differentiation of intestinal Foxp3^+^-Tregs in addition to the production of IL-10 and TGF-*β* that regulate the functions of intestinal myeloid cells [54]. Some probiotic strains, for instance, *Lactobacillus acidophilus*, improve intestinal inflammation by modulating the balance of Th17 and Treg cells [55, 56]. SCFAs, which are an energy source for gut epithelial cells, serve an anti-inflammatory function by inhibiting HDACs in Tregs through G-protein-coupled receptors (GPRs). SCFAs can also promote naïve T cell differentiation into both effector T cells and Treg cells depending on the cytokine milieu, which is dependent on direct suppression of HDACs, independent of GPR41 or GPR43. Plasticity features of Foxp3^+^-Treg cells facilitate their acquisition of an effector T cell phenotype in highly virulent or inflammatory contexts, and their fates are controlled by pathogenic effector T cells [57].

ROR*γ*t^+^ Tregs are a distinct Treg population in the colon. The expression of transcription factor ROR*γ*t is induced by gut microbiota. The proportion of ROR*γ*t^+^ Tregs is much lower in GF mice than in their conventionally raised specific-pathogen-free (SPF) counterparts [58]. The context specificity of ROR*γ* results in significantly different outcomes, even in closely related cell types, which are consistent with their involvement in a range of immunological and nonimmunological processes. A recent study has demonstrated that weaning immune reaction to microbiota is associated with the generation of ROR*γ*t^+^-Treg cells that decreases the susceptibility to allergic inflammation, colitis, and cancer later in life [59]. Moreover, in colorectal cancer (CAC) patients with IBD background, ROR*γ*t-expressing tumor-infiltrating Treg cells sustain tumor growth in a transcription factor FoxO3-dependent manner [60]. However, the frequency of ROR*γ*t^+^ Tregs correlates with the colitis score when momocolonized with different microbes in trinitrobenzenesulfonic acid- (TNBS-) challenged GF mice [58]. Foxp3^+^ROR*γ*t^+^ T cells are an important subset of effector Treg cells of the intestinal immune system that displays features of both Tregs and Th17 cells. They accumulate in the LP of IBD patients that have enhanced immunosuppressive capacity as compared with Foxp3^+^ROR*γ*t^−^ Tregs during gut-specific immune responses [61]. However, a contradictory finding indicates that inactivation of ROR*γ*t in Treg cells has a minor effect on the balance of bacteria-specific Treg and Th17 cells and does not lead to inflammation. In addition, the expression of the transcription factor c-Maf is required for the terminal differentiation and function of ROR*γ*t^+^ Treg cells in inhibiting intestinal Th17 responses [62].

The close relationship between Th17 and Treg cells marks them important in modulating the immune responses and maintaining immune homeostasis. Molecular mediators that induce ROR*γ*t^+^ Treg development remain obscure. It is likely that SCFA is one of the mediators, and ROR*γ*t^+^ induction in Th17 and colonic Tregs may follow a different pathway [58]. Currently, availability of small-molecule inhibitors, such as TAK-828F, or activators of metabolic enzymes has made it possible to manipulate the metabolism of T cells and shift the Th17/Treg cell balance, providing a novel therapeutic option for the treatment of inflammatory and immune diseases [27, 63].

### 3.3. Regulation of Intestinal T Follicular Helper (Tfh) Cells by Microbiota

Tfh has been identified as the chief cell subpopulation regulating B cells in germinal centers (GCs) that promotes high-affinity antibody production. Tfh development is deficient in GF mice and can be restored when fed by toll-like receptor-2 (TLR2) agonists via activating intrinsic MyD88 signaling [64]. Ablation of Tfh cells results in reduced amount of PPs, IgG1, and GC B cells. And significantly changes the gut microbiome composition [26]. Thus, Tfh cell activity is important for the generation of a diverse microbiota community in the gut. IL-21 produced by Tfh cells in PPs is essential in driving the GC reaction and high-affinity sIgA production in the small intestine [26]. IL-21R-deficient mice exhibit a significant decrease in IgA^+^ plasmablasts and plasma cells, in response to SFB in the small intestine [65]. SFB can also drive differentiation of PP Tfh cells and egress into systemic sites, boosting systemic Tfh cell responses and autoantibody production that exacerbates arthritis [66]. Microbiota-derived extracellular ATP (eATP) limits Tfh cell expansion and GC reaction in the PPs via P2X7, regulates Tfh cell abundance, and affects high-affinity sIgA response against intestinal colonizing bacteria that leads to enteropathogenic infection [67, 68]. Thus, microbiota-derived eATP is an important signaling molecule, which can be a further modulation target of the intestinal immunity against intestinal bacteria. Several transcription factors are involved in the divergent functions of Tfh. Activating transcription factor 3 (ATF3) can protect against colitis by regulating Tfhs in the gut [69]. Interferon regulatory factor 8- (IRF8-) regulated Tfhs can function as pathogenic mediators of colitis in IBD, which is independent of B cells [70]. Transcription factor c-Maf is expressed early in Tfh cell precursors and identified as a regulator in the differentiation of Tfh cells in a cell-autonomous fashion [71].

Antibodies produced in the GC need precisely targeting of foreign pathogens while limiting excessive inflammation and autoimmunity for maintaining normal intestinal circumstances. T follicular regulatory (Tfr) cells, a recently identified cell subset, can migrate to the GC and inhibit Tfh-mediated B cell activation and Ig production. Deep understanding of Tfr cell function has potential to provide new insights into developing more effective vaccine strategies and new methods to treat antibody-mediated diseases [72].

## 4. Induction of Antivirus and Anticancer Immunity of Intestinal CD8^+^ T Cells by Gut Microbiota and Metabolites

The effects of intestinal microbiota and metabolites on intestinal CD8^+^ T cells functions are relatively uncharacterized. Previous studies have shown that recolonization of VSL#3 and *L. johnsonii* results in restoration of CD4^+^ and CD8^+^ T cell population in the small and large intestinal LP and mesenteric lymph nodes (MLNs) or partial restoration upon FMT after broad-spectrum antibiotic therapy [8, 10]. SCFAs, especially butyrate, directly modulate IFN-*γ* and granzyme B gene expression of CD8^+^ CTLs and IL-17-producing CD8^+^ T cells (Tc17 cells) in MLNs, which is mediated by inhibition of HDACs, independent of GPR41 and GPR43. Moreover, this influence is similar to the effects exerted by the pan-HDAC inhibitors trichostatin A (TSA) and sodium valproate [73]. It is shown that a consortium of 11 bacterial strains obtained from healthy human donor feces is capable of robustly inducing IFN-*γ*-producing CD8^+^ T cells in the intestine. Their colonization enhances both host resistance to *Listeria monocytogenes* infection and the therapeutic efficacy of ICIs in syngeneic tumor models [74].

IELs that distribute along the intestinal epithelium are a large and diverse population of lymphoid cells, which are classified into TCR^+^ and TCR^−^ subsets. The former is similar to conventional T cells, whereas the latter functions resembling ILCs. Natural TCR^+^ IELs include TCR*αβ*^+^ or TCR*γδ*^+^ T cells. In the small intestine of mice, 50%–60% of the IELs are TCR*γδ*^+^ cells, whereas in humans, CD8*αβ*^+^ IELs are the predominant population. There exists an intricate interaction between IEL subsets and IECs in the intestine, and other immune cells outside IECs [75]. Colonization of commensal microbiota in early life is important for the development of diet-induced CD8*αβ*^+^ IELs [76]. Intestinal microbiota, such as *Bacteroidales*, recruits IL-6-producing IELs promoting intestinal epithelial proliferation, contributing to barrier integrity [77]. In antibiotic-treated and GF mice, the proportion and absolute number of CD8*αβ*^+^ IELs drop significantly. Microarray analysis has revealed that CD8*αβ*^+^ IELs expressed a series of genes encoding potent AMPs, which is supported by an antimicrobial-activity assay [78]. *γδ*^+^ IELs can promptly migrate to and remain localized near IEC lumen in direct contact with bacteria in the case of pathogen invasion within hours, suggesting their essential role in early host defense against pathogen invasion [79]. Their activation is dependent on microbe-activated secretion of intrinsic MyD88 by IECs [80]. Assay for Transposase-Accessible Chromatin with high-throughput sequencing (ATAC-seq) of intestinal *αβ*^+^ and *γδ*^+^ IELs has disclosed that their enhancer-associated transcription factors, signaling networks, and metabolic pathways are affected by the microbiota [12].

In mice, the small intestine contains CD4^+^CD8*αα*^+^ double-positive IELs (DP IELs), which have regulatory functions originated from intestinal CD4^+^ T cells through downstream of the transcriptional factor Thpok. Uncommon in human adults, the differentiation of DP IELs depends on microbial colonization because they are not present in GF mice [81]. *Lactobacillus reuteri* may reprogram intraepithelial CD4^+^ T cells into DP IELs in both GF mice and conventionally raised counterparts lacking these cells. Though not shaping the DP-IEL-TCR repertoire, it generates indole derivatives of tryptophan activating the aryl-hydrocarbon receptor that downregulates Thpok and promoting CD4^+^ T cell differentiation into DP IELs. Thus, *L. reuteri*, together with a tryptophan-rich diet, results in the reprograming of IELs into cells of regulatory phenotype [82].

## 5. Induction of sIgA Production by Gut Microbiota and Metabolites

In the gut lumen, sIgA serves as a first-line barrier protecting the epithelium from invasion of pathogens and toxins by promoting mutualistic microbe colonization and neutralization of invasive pathogens. SIgA coats bacteria and other targets, thus preventing them from direct contact with the host intestinal immune system. A high level of sIgA production results from the interactions of intestinal Tfhs and B cells in the GCs. Due to sustained microbial antigen exposure, intestinal lymphoid tissues generate flora-reactive IgA-producing B cells [83]. Maturation of isolated lymphoid follicles (ILFs) into large B cell clusters requires the detection of bacteria by pattern-recognition receptors (PRRs) [84]. Peptidoglycan from gram-negative bacteria is necessary for the induction of ILFs in mice through recognition by the nucleotide oligomerization domain 1 (NOD1) receptor in epithelial cells and other signaling through the chemokine receptor CCR6 [83].

Intestinal microbiota influences not only the accumulation of sIgA-producing plasma cells but also the diversity of IgA in intestine lymphocyte tissue which has been demonstrated in gnotobiotic mice colonized with defined microbial consortia [85]. IgA directly participate in shaping the microbial community landscape. During infection, sIgA-coated pathogens are cleared via a process called immune exclusion [86, 87]. The diversity of IgA on the mammalian intestinal surface is paralleled with the intestinal taxa diversity [88]. PPs are essential sites for the TD IgA generation, contributing not only to the generation of somatically mutated gut antigen-specific IgAs production but also to the diversification of nonspecific antigen of the B cell repertoire [89, 90]. Moreover, PP can be substituted as the inductive sites for IgA production with the stimulation of specific immunostimulatory or pathogenic bacteria, such as SFB [84]. B cell development in GALT is also driven by super-antigen-like molecules, such as spores of *Bacillus subtilis* and other species of *Bacillus* [91]. SCFAs can promote IgA production through regulating the metabolism and gene expression in B cells independent of T cells [92, 93].

SIgA in response to the commensal microbiota is produced through a T cell-independent (TI) mechanism which has been confirmed in TCR*αβ* and TCR*γδ* T cell-deficient mice. These processes may not require PP-organized B cell follicles, which are specific for particular bacterial species. The intestinal TI-IgAs are highly stable antibody repertoires that perform a dual role in the protection against invading pathogens and the regulation of the composition of nonpathogenic microbial communities [94, 95].

### 5.1. Interactions of sIgA with Other Immune Cells and Tolerance Induction

Plasmacytoid DCs (pDCs) are essential for the induction of plasma cells in response to oral immunization and noninfectious antibody responses with the production of TD or TI-IgA in the intestine [96, 97]. PP IgA production, affinity maturation, and class switch recombination require PP subepithelial dome DC–B cell interactions, which are dependent on the secretion of TGF-*β* by ILCs [89]. The bacteria-loaded DCs induce the differentiation of B cells into IgA^+^ plasma cells from lymphoid structures to LP, dimeric IgA secretion, and restrict the mucosal immunity locally [98]. In turn, sIgA-coated commensal bacteria contribute mucosal DCs towards tolerogenic profiles [99] and Treg cell expansion and IL-10 production, which dampen mucosal and systemic autoimmunity [100].

Human gut bacterial taxa targeted by IgA in the setting of barrier dysfunction result in intestinal pathology that may be attributed to the adhesion of these taxa to intestinal microfold (M) cells, a subset of IECs residing in the region of the epithelium covering PPs. For instance, the sIgA-coated *Clostridium difficile* [101] and adherent-invasive *E. coli* [102], which are located in proximity to the intestinal epithelium, elicit a Th17-cell-dependent sIgA-mediated response. The precise link of sIgA diversification and specific antigenic functions in modulating the microbiota composition, location, and metabolism are yet to be elucidated [88]. A novel technology of 16S ribosomal RNA (rRNA) gene sequencing of immunoglobulin A- (IgA-) coated bacteria (IgA-SEQ) may be helpful in the isolation and identification of unique colitogenic intestinal bacteria [103].

**Figure 1:**
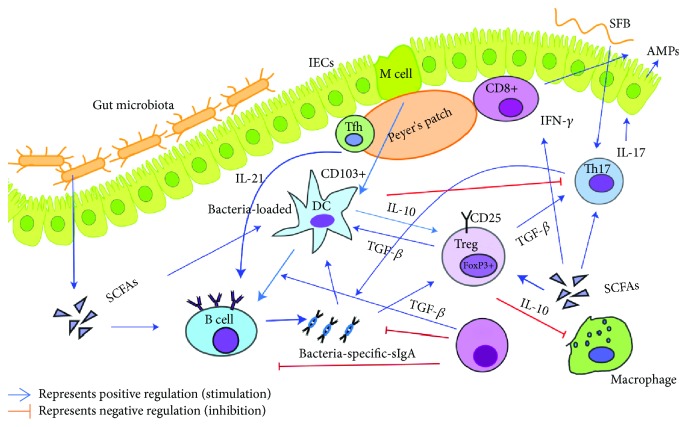
The intestinal microbiota modulate the intestinal mucosal adaptive immunity and interactions between immune cells. The intestinal microbiota, their components, and metabolites contribute to the regulation of innate and adaptive immune responses during homeostasis and dysbiosis states. Under homeostatic conditions, microbiota and microbial metabolites contribute to the intestinal immunological tolerance *via* Tregs and sIgA. Tolerogenic DCs contribute to the induction of Tregs and sIgA production by IL-10 secretion. Bacterial components such as SCFAs are potent inducers of Tregs and B cells and promote IFN-*γ* production from CD8^+^ T cells. Microbiota, SCFAs, and IL-21 secreted from the Tfh in the PP contribute to the secretion of bacteria-specific sIgA. Segmented filamentous bacteria induce SFB-specific Th17 cell production. Tregs modulate DCs and Th17 cells mediated by TGF-*β*. DCs and sIgA negatively regulate the function of Th17 cells.

In brief, commensal microbiota trains and regulates the development and maturation of the intestinal adaptive immune system. Under a steady state, the mucosal adaptive immune cells, especially Tregs and Tfh, and sIgA coordinate with IECs, DCs, and ILCs contributing to a tolerogenic state of intestinal ecological environment, maintaining intestinal homeostasis and mutualistic relationship between the host and the microbiota, thus preserving the ability of eliciting an efficient immune response to infectious agents. In the case of dysbiosis, the equilibrium is disturbed that leads to chronic inflammatory and autoimmune pathology. Mechanisms of intestinal microbiota modulation of intestinal adaptive immunity are depicted in Figure 1.

## 6. Conclusions and Perspectives

Under a steady state, commensal microbiota and intestinal adaptive immune cells and sIgA coordinate to contribute towards tolerance for symbiotic bacteria and mutualistic relationship between the host and the microbiota. This ultimately results in maintenance of intestinal homeostasis and the retention of the ability to elicit an efficient immune response to invading pathogenic agents. When this equilibrium relationship is disturbed, dysbiosis and intestinal immunological abnormalities develop leading to chronic local and systemic inflammatory and autoimmune disorders. Identifying effector microorganisms, derived molecules, and metabolites that causally affect an immune phenotype holds the key for deciphering the underlying mechanisms and the development of microbiota-based therapeutics [104–106]. The present-day widely applied molecular sequencing-based methods do not allow for the isolation of specific organisms, hindering the exploration of the association of their functional roles with host-specific immune parameters and definite disease phenotype. In the near future, database-centered predictions of strain-media, IgA-SEQ, engineered bacteria, high-throughput culturing, and microfluidic assays [106–108] may help to isolate uncultivated species of interest [109, 110]. These technical advances may thus provide utmost probabilities for exploring the mechanisms of microbiota-host interactions improving diagnostic and therapeutic applications.

## References

[1] Levy M., Kolodziejczyk A. A., Thaiss C. A., Elinav E. (2017). Dysbiosis and the immune system. *Nature Reviews. Immunology*.

[2] Kabat A. M., Srinivasan N., Maloy K. J. (2014). Modulation of immune development and function by intestinal microbiota. *Trends in Immunology*.

[3] Gunther C., Josenhans C., Wehkamp J. (2016). Crosstalk between microbiota, pathogens and the innate immune responses. *International Journal of Medical Microbiology*.

[4] Honda K., Littman D. R. (2016). The microbiota in adaptive immune homeostasis and disease. *Nature*.

[5] Agace W. W., McCoy K. D. (2017). Regionalized development and maintenance of the intestinal adaptive immune landscape. *Immunity*.

[6] Nguyen Q. N., Himes J. E., Martinez D. R., Permar S. R. (2016). The impact of the gut microbiota on humoral immunity to pathogens and vaccination in early infancy. *PLOS Pathogens*.

[7] Meisel M., Mayassi T., Fehlner-Peach H. (2017). Interleukin-15 promotes intestinal dysbiosis with butyrate deficiency associated with increased susceptibility to colitis. *The ISME Journal*.

[8] Chiu C. Y., Chan Y. L., Tsai M. H., Wang C. J., Chiang M. H., Chiu C. C. (2019). Gut microbial dysbiosis is associated with allergen-specific IgE responses in young children with airway allergies. *World Allergy Organization Journal*.

[9] Ekmekciu I., von Klitzing E., Fiebiger U. (2017). The probiotic compound VSL#3 modulates mucosal, peripheral, and systemic immunity following murine broad-spectrum antibiotic treatment. *Frontiers in Cellular and Infection Microbiology*.

[10] Ren Y. D., Ye Z. S., Yang L. Z. (2017). Fecal microbiota transplantation induces hepatitis B virus e-antigen (HBeAg) clearance in patients with positive HBeAg after long-term antiviral therapy. *Hepatology*.

[11] Ekmekciu I., von Klitzing E., Neumann C. (2017). Fecal microbiota transplantation, commensal Escherichia coli and Lactobacillus johnsonii strains differentially restore intestinal and systemic adaptive immune cell populations following broad-spectrum antibiotic treatment. *Frontiers in Microbiology*.

[12] Semenkovich N. P., Planer J. D., Ahern P. P., Griffin N. W., Lin C. Y., Gordon J. I. (2016). Impact of the gut microbiota on enhancer accessibility in gut intraepithelial lymphocytes. *Proceedings of the National Academy of Sciences*.

[13] Xu T., Stewart K. M., Wang X. (2017). Metabolic control of TH17 and induced Treg cell balance by an epigenetic mechanism. *Nature*.

[14] Chen L., Sun M., Wu W. (2019). Microbiota metabolite butyrate differentially regulates Th1 and Th17 cells’ differentiation and function in induction of colitis. *Inflammatory Bowel Diseases*.

[15] Roy S., Trinchieri G. (2017). Microbiota: a key orchestrator of cancer therapy. *Nature Reviews. Cancer*.

[16] Pitt J. M., Vetizou M., Waldschmitt N. (2016). Fine-tuning cancer immunotherapy: optimizing the gut microbiome. *Cancer Research*.

[17] Routy B., Le Chatelier E., Derosa L. (2018). Gut microbiome influences efficacy of PD-1–based immunotherapy against epithelial tumors. *Science*.

[18] Sivan A., Corrales L., Hubert N. (2015). Commensal Bifidobacterium promotes antitumor immunity and facilitates anti-PD-L1 efficacy. *Science*.

[19] Matson V., Fessler J., Bao R. (2018). The commensal microbiome is associated with anti-PD-1 efficacy in metastatic melanoma patients. *Science*.

[20] Vetizou M., Pitt J. M., Daillere R. (2015). Anticancer immunotherapy by CTLA-4 blockade relies on the gut microbiota. *Science*.

[21] Chairatana P., Nolan E. M. (2017). Defensins, lectins, mucins, and secretory immunoglobulin A: microbe-binding biomolecules that contribute to mucosal immunity in the human gut. *Critical Reviews in Biochemistry and Molecular Biology*.

[22] Melo-Gonzalez F., Kammoun H., Evren E. (2019). Antigen-presenting ILC3 regulate T cell–dependent IgA responses to colonic mucosal bacteria. *The Journal of Experimental Medicine*.

[23] Ladinsky M. S., Araujo L. P., Zhang X. (2019). Endocytosis of commensal antigens by intestinal epithelial cells regulates mucosal T cell homeostasis. *Science*.

[24] Zhao Q., Elson C. O. (2018). Adaptive immune education by gut microbiota antigens. *Immunology*.

[25] Feng T., Elson C. O. (2011). Adaptive immunity in the host-microbiota dialog. *Mucosal Immunology*.

[26] Jones L., Ho W. Q., Ying S. (2016). A subpopulation of high IL-21-producing CD4^+^ T cells in Peyer’s Patches is induced by the microbiota and regulates germinal centers. *Scientific Reports*.

[27] Sun L., Fu J., Zhou Y. (2017). Metabolism controls the balance of Th17/T-regulatory cells. *Frontiers in Immunology*.

[28] Kespohl M., Vachharajani N., Luu M. (2017). The microbial metabolite butyrate induces expression of Th1-associated factors in CD4^+^ T Cells. *Frontiers in Immunology*.

[29] Britton G. J., Contijoch E. J., Mogno I. (2019). Microbiotas from humans with inflammatory bowel disease alter the balance of gut Th17 and ROR*γ*t^+^ regulatory T cells and exacerbate colitis in mice. *Immunity*.

[30] Han L., Jin H., Zhou L. (2018). Intestinal microbiota at engraftment influence acute graft-versus-host disease via the Treg/Th17 balance in allo-HSCT recipients. *Frontiers in Immunology*.

[31] Bettelli E., Carrier Y., Gao W. (2006). Reciprocal developmental pathways for the generation of pathogenic effector TH17 and regulatory T cells. *Nature*.

[32] Romano M., Tung S. L., Smyth L. A., Lombardi G. (2017). Treg therapy in transplantation: a general overview. *Transplant International*.

[33] Weaver C. T., Elson C. O., Fouser L. A., Kolls J. K. (2013). The Th17 pathway and inflammatory diseases of the intestines, lungs, and skin. *Annual Review of Pathology*.

[34] Stockinger B., Omenetti S. (2017). The dichotomous nature of T helper 17 cells. *Nature Reviews. Immunology*.

[35] Hirota K., Turner J. E., Villa M. (2013). Plasticity of Th17 cells in Peyer’s patches is responsible for the induction of T cell-dependent IgA responses. *Nature Immunology*.

[36] Zhao M., Tan Y., Peng Q. (2018). IL-6/STAT3 pathway induced deficiency of RFX1 contributes to Th17-dependent autoimmune diseases via epigenetic regulation. *Nature Communications*.

[37] Atarashi K., Tanoue T., Ando M. (2015). Th17 cell induction by adhesion of microbes to intestinal epithelial cells. *Cell*.

[38] Yang Y., Torchinsky M. B., Gobert M. (2014). Focused specificity of intestinal TH17 cells towards commensal bacterial antigens. *Nature*.

[39] Yi J., Jung J., Han D., Surh C. D., Lee Y. J. (2019). Segmented filamentous bacteria induce divergent populations of antigen-specific CD4 T cells in the small intestine. *Molecules and Cells*.

[40] Ueno A., Jeffery L., Kobayashi T., Hibi T., Ghosh S., Jijon H. (2018). Th17 plasticity and its relevance to inflammatory bowel disease. *Journal of Autoimmunity*.

[41] Wong S. H., Zhao L., Zhang X. (2017). Gavage of fecal samples from patients with colorectal cancer promotes intestinal carcinogenesis in germ-free and conventional mice. *Gastroenterology*.

[42] Hurtado C. G., Wan F., Housseau F., Sears C. L. (2018). Roles for interleukin 17 and adaptive immunity in pathogenesis of colorectal cancer. *Gastroenterology*.

[43] Gopalakrishnan V., Spencer C. N., Nezi L. (2018). Gut microbiome modulates response to anti-PD-1 immunotherapy in melanoma patients. *Science*.

[44] Li J., Doty A. L., Tang Y. (2017). Enrichment of IL‐17A^+^IFN‐*γ*^+^ and IL‐22^+^IFN‐*γ*^**+**^ T cell subsets is associated with reduction of NKp44^+^ILC3s in the terminal ileum of Crohn’s disease patients. *Clinical and Experimental Immunology*.

[45] Zhang Y. S., Xin D. E., Wang Z. (2019). STAT4 activation by leukemia inhibitory factor confers a therapeutic effect on intestinal inflammation. *The EMBO Journal*.

[46] Hu W., Pasare C. (2013). Location, location, location: tissue-specific regulation of immune responses. *Journal of Leukocyte Biology*.

[47] Geginat J., Paroni M., Kastirr I., Larghi P., Pagani M., Abrignani S. (2016). Reverse plasticity: TGF-*β* and IL-6 induce Th1-to-Th17-cell transdifferentiation in the gut. *European Journal of Immunology*.

[48] Rizzo A., de Mare V., Rocchi C. (2014). Smad7 induces plasticity in tumor-infiltrating Th17 cells and enables TNF-alpha-mediated killing of colorectal cancer cells. *Carcinogenesis*.

[49] Sujino T., London M., Hoytema van Konijnenburg D. P. (2016). Tissue adaptation of regulatory and intraepithelial CD4^**+**^ T cells controls gut inflammation. *Science*.

[50] Kraj P., Ignatowicz L. (2018). The mechanisms shaping the repertoire of CD4(+) Foxp3(+) regulatory T cells. *Immunology*.

[51] Atarashi K., Tanoue T., Shima T. (2011). Induction of colonic regulatory T cells by indigenous Clostridium species. *Science*.

[52] Atarashi K., Tanoue T., Oshima K. (2013). Treg induction by a rationally selected mixture of Clostridia strains from the human microbiota. *Nature*.

[53] Round J. L., Mazmanian S. K. (2010). Inducible Foxp3+ regulatory T-cell development by a commensal bacterium of the intestinal microbiota. *Proceedings of the National Academy of Sciences of the United States of America*.

[54] Kayama H., Takeda K. (2012). Regulation of intestinal homeostasis by innate and adaptive immunity. *International Immunology*.

[55] Park J., Kim M., Kang S. G. (2015). Short-chain fatty acids induce both effector and regulatory T cells by suppression of histone deacetylases and regulation of the mTOR-S6K pathway. *Mucosal Immunology*.

[56] Park J. S., Choi J. W., Jhun J. (2018). *Lactobacillus acidophilus* improves intestinal inflammation in an acute colitis mouse model by regulation of Th17 and Treg cell balance and fibrosis development. *Journal of Medicinal Food*.

[57] Oldenhove G., Bouladoux N., Wohlfert E. A. (2009). Decrease of Foxp3+ Treg cell number and acquisition of effector cell phenotype during lethal infection. *Immunity*.

[58] Sefik E., Geva-Zatorsky N., Oh S. (2015). Individual intestinal symbionts induce a distinct population of ROR*γ*^+^ regulatory T cells. *Science*.

[59] Al Nabhani Z., Dulauroy S., Marques R. (2019). A weaning reaction to microbiota is required for resistance to immunopathologies in the adult. *Immunity*.

[60] Rizzo A., di Giovangiulio M., Stolfi C. (2018). ROR*γ*t-expressing Tregs drive the growth of colitis-associated colorectal cancer by controlling IL6 in dendritic cells. *Cancer Immunology Research*.

[61] Yang B. H., Hagemann S., Mamareli P. (2016). Foxp3^+^ T cells expressing ROR*γ*t represent a stable regulatory T-cell effector lineage with enhanced suppressive capacity during intestinal inflammation. *Mucosal Immunology*.

[62] Neumann C., Blume J., Roy U. (2019). c-Maf-dependent Treg cell control of intestinal TH17 cells and IgA establishes host-microbiota homeostasis. *Nature Immunology*.

[63] Shibata A., Uga K., Sato T. (2018). Pharmacological inhibitory profile of TAK-828F, a potent and selective orally available ROR*γ*t inverse agonist. *Biochemical Pharmacology*.

[64] Kubinak J. L., Petersen C., Stephens W. Z. (2015). MyD88 signaling in T cells directs IgA-mediated control of the microbiota to promote health. *Cell Host & Microbe*.

[65] Cho H., Jaime H., de Oliveira R. P. (2019). Defective IgA response to atypical intestinal commensals in IL-21 receptor deficiency reshapes immune cell homeostasis and mucosal immunity. *Mucosal Immunology*.

[66] Teng F., Klinger C. N., Felix K. M. (2016). Gut microbiota drive autoimmune arthritis by promoting differentiation and migration of Peyer’s patch T follicular helper cells. *Immunity*.

[67] Perruzza L., Gargari G., Proietti M. (2017). T follicular helper cells promote a beneficial gut ecosystem for host metabolic homeostasis by sensing microbiota-derived extracellular ATP. *Cell Reports*.

[68] Proietti M., Perruzza L., Scribano D. (2019). ATP released by intestinal bacteria limits the generation of protective IgA against enteropathogens. *Nature Communications*.

[69] Cao Y., Yang Q., Deng H. (2019). Transcriptional factor ATF3 protects against colitis by regulating follicular helper T cells in Peyer’s patches. *Proceedings of the National Academy of Sciences of the United States of America*.

[70] Zhang R., Qi C. F., Hu Y. (2019). T follicular helper cells restricted by IRF8 contribute to T cell-mediated inflammation. *Journal of Autoimmunity*.

[71] Andris F., Denanglaire S., Anciaux M., Hercor M., Hussein H., Leo O. (2017). The transcription factor c-Maf promotes the differentiation of follicular helper T cells. *Frontiers in Immunology*.

[72] Sage P. T., Sharpe A. H. (2016). T follicular regulatory cells. *Immunological Reviews*.

[73] Luu M., Weigand K., Wedi F. (2018). Regulation of the effector function of CD8^+^ T cells by gut microbiota-derived metabolite butyrate. *Scientific Reports*.

[74] Tanoue T., Morita S., Plichta D. R. (2019). A defined commensal consortium elicits CD8 T cells and anti-cancer immunity. *Nature*.

[75] Olivares-Villagomez D., Van Kaer L. (2018). Intestinal intraepithelial lymphocytes: sentinels of the mucosal barrier. *Trends in Immunology*.

[76] Jung J., Surh C. D., Lee Y. J. (2019). Microbial colonization at early life promotes the development of diet-induced CD8*αβ* intraepithelial T cells. *Molecules and Cells*.

[77] Kuhn K. A., Schulz H. M., Regner E. H. (2018). Bacteroidales recruit IL-6-producing intraepithelial lymphocytes in the colon to promote barrier integrity. *Mucosal Immunology*.

[78] Chen B., Ni X., Sun R. (2018). Commensal bacteria-dependent CD8*αβ*^+^ T cells in the intestinal epithelium produce antimicrobial peptides. *Frontiers in Immunology*.

[79] Edelblum K. L., Singh G., Odenwald M. A. (2015). *γδ* intraepithelial lymphocyte migration limits transepithelial pathogen invasion and systemic disease in mice. *Gastroenterology*.

[80] Ismail A. S., Severson K. M., Vaishnava S. (2011). *γδ* intraepithelial lymphocytes are essential mediators of host–microbial homeostasis at the intestinal mucosal surface. *Proceedings of the National Academy of Sciences of the United States of America*.

[81] Wu C., Sartor R. B., Huang K., Tonkonogy S. L. (2016). Transient activation of mucosal effector immune responses by resident intestinal bacteria in normal hosts is regulated by interleukin-10 signalling. *Immunology*.

[82] Cervantes-Barragan L., Chai J. N., Tianero M. D. (2017). *Lactobacillus reuteri* induces gut intraepithelial CD4^+^CD8*αα*^+^ T cells. *Science*.

[83] Bouskra D., Brézillon C., Bérard M. (2008). Lymphoid tissue genesis induced by commensals through NOD1 regulates intestinal homeostasis. *Nature*.

[84] Buettner M., Lochner M. (2016). Development and function of secondary and tertiary lymphoid organs in the small intestine and the colon. *Frontiers in Immunology*.

[85] Moon C., Baldridge M. T., Wallace M. A., Burnham C. A. D., Virgin H. W., Stappenbeck T. S. (2015). Vertically transmitted faecal IgA levels determine extra-chromosomal phenotypic variation. *Nature*.

[86] Roche A. M., Richard A. L., Rahkola J. T., Janoff E. N., Weiser J. N. (2015). Antibody blocks acquisition of bacterial colonization through agglutination. *Mucosal Immunology*.

[87] Pabst O. (2012). New concepts in the generation and functions of IgA. *Nature Reviews. Immunology*.

[88] Macpherson A. J., Yilmaz B., Limenitakis J. P., Ganal-Vonarburg S. C. (2018). IgA function in relation to the intestinal microbiota. *Annual Review of Immunology*.

[89] Reboldi A., Arnon T. I., Rodda L. B., Atakilit A., Sheppard D., Cyster J. G. (2016). IgA production requires B cell interaction with subepithelial dendritic cells in Peyer’s patches. *Science*.

[90] Reboldi A., Cyster J. G. (2016). Peyer’s patches: organizing B-cell responses at the intestinal frontier. *Immunological Reviews*.

[91] Severson K. M., Mallozzi M., Driks A., Knight K. L. (2010). B cell development in GALT: role of bacterial superantigen-like molecules. *Journal of Immunology*.

[92] Kim M., Qie Y., Park J., Kim C. H. (2016). Gut microbial metabolites fuel host antibody responses. *Cell Host & Microbe*.

[93] Wu W., Sun M., Chen F. (2017). Microbiota metabolite short-chain fatty acid acetate promotes intestinal IgA response to microbiota which is mediated by GPR43. *Mucosal Immunology*.

[94] Bemark M., Boysen P., Lycke N. Y. (2012). Induction of gut IgA production through T cell-dependent and T cell-independent pathways. *Annals of the New York Academy of Sciences*.

[95] Allman D., Wilmore J. R., Gaudette B. T. (2019). The continuing story of T-cell independent antibodies. *Immunological Reviews*.

[96] Tezuka H., Abe Y., Asano J. (2011). Prominent role for plasmacytoid dendritic cells in mucosal T cell-independent IgA induction. *Immunity*.

[97] Moro-Sibilot L., This S., Blanc P. (2016). Plasmacytoid dendritic cells are dispensable for noninfectious intestinal IgA responses in vivo. *European Journal of Immunology*.

[98] Macpherson A. J., Uhr T. (2004). Induction of protective IgA by intestinal dendritic cells carrying commensal bacteria. *Science*.

[99] Diana J., Moura I. C., Vaugier C. (2013). Secretory IgA induces tolerogenic dendritic cells through SIGNR1 dampening autoimmunity in mice. *Journal of Immunology*.

[100] Mikulic J., Longet S., Favre L., Benyacoub J., Corthesy B. (2017). Secretory IgA in complex with *Lactobacillus rhamnosus*potentiates mucosal dendritic cell-mediated Treg cell differentiation via TLR regulatory proteins, RALDH2 and secretion of IL-10 and TGF-*β*. *Cellular & Molecular Immunology*.

[101] Džunková M., Moya A., Vázquez-Castellanos J. F. (2016). Active and secretory IgA-coated bacterial fractions elucidate dysbiosis in *Clostridium difficile* infection. *mSphere*.

[102] Viladomiu M., Kivolowitz C., Abdulhamid A. (2017). IgA-coated E. coli enriched in Crohn’s disease spondyloarthritis promote T_H_17-dependent inflammation. *Science Translational Medicine*.

[103] Palm N. W., de Zoete M. R., Cullen T. W. (2014). Immunoglobulin A coating identifies colitogenic bacteria in inflammatory bowel disease. *Cell*.

[104] Geva-Zatorsky N., Sefik E., Kua L. (2017). Mining the human gut microbiota for immunomodulatory organisms. *Cell*.

[105] Skelly A. N., Sato Y., Kearney S., Honda K. (2019). Mining the microbiota for microbial and metabolite-based immunotherapies. *Nature Reviews. Immunology*.

[106] Rajpoot M., Sharma A. K., Sharma A., Gupta G. K. (2018). Understanding the microbiome: emerging biomarkers for exploiting the microbiota for personalized medicine against cancer. *Seminars in Cancer Biology*.

[107] Rojo D., Méndez-García C., Raczkowska B. A. (2017). Exploring the human microbiome from multiple perspectives: factors altering its composition and function. *FEMS Microbiology Reviews*.

[108] Riglar D. T., Silver P. A. (2018). Engineering bacteria for diagnostic and therapeutic applications. *Nature Reviews. Microbiology*.

[109] Oberhardt M. A., Zarecki R., Gronow S. (2015). Harnessing the landscape of microbial culture media to predict new organism–media pairings. *Nature Communications*.

[110] Lau J. T., Whelan F. J., Herath I. (2016). Capturing the diversity of the human gut microbiota through culture-enriched molecular profiling. *Genome Medicine*.

